# Supplementary material for: Translational rapid ultraviolet-excited sectioning tomography for whole-organ multicolor imaging with real-time molecular staining
Source: eLife. 2022 Nov 4;11:e81015. doi: 10.7554/eLife.81015 (PMC9671503; doi:10.7554/eLife.81015)
Supplement: Supplementary file 1. — (a) Parameters of the staining solutions and the white balance. Concentrations of the staining solutions and the rates for adding additional dyes into the water tank to maintain the same concentration of the staining solutions. Note: the control of pumping rate is not necessarily needed and can be different depending on the size of the specimen and volume of the staining solutions in the water tank. The white balance (red/blue) parameters of the color camera (DS-Fi3, Nikon Corp.) in TRUST are also provided as a reference. (b) Comparison of TRUST with other whole-organ imaging modalities. The imaging speed of each modality is calculated based on the reported literature for whole mouse brain imaging, including serial two-photon tomography (STPT) Ragan et al., 2012, microtomy-assisted photoacoustic microscopy (mPAM) Wong et al., 2012, micro-optical sectioning tomography (MOST) Li et al., 2010, fluorescence micro-optical sectioning tomography (fMOST) Gong et al., 2013, light-sheet fluorescence microscopy (LSFM) Matsumoto et al., 2019, and wide-field large-volume tomography (WVT) Gong et al., 2016. [file elife-81015-supp1.docx]

**Translational Rapid Ultraviolet-excited Sectioning Tomography for Whole-organ Multicolor Imaging with Real-time Molecular Staining**

**Wentao Yu, Lei Kang, Victor T. C. Tsang, Yan Zhang, Ivy H. M. Wong, Terence T. W. Wong^*^**

*Translational and Advanced Bioimaging Laboratory, Department of Chemical and Biological Engineering, The Hong Kong University of Science and Technology, Kowloon, Hong Kong, China*

**^*^***Corresponding author:* [*ttwwong@ust.hk*](mailto:ttwwong@ust.hk)

The Supplementary file includes:

**Supplementary file 1a.** Parameters of the staining solutions and the white balance.

**Supplementary file 1b.** Comparison of TRUST with other whole-organ imaging modalities.

**Supplementary file 1a. Parameters of the staining solutions and the white balance.** Concentrations of the staining solutions and the rates for adding additional dyes into the water tank to maintain the same concentration of the staining solutions. Note: the control of pumping rate is not necessarily needed and can be different depending on the size of the specimen and volume of the staining solutions in the water tank. The white balance (red/blue) parameters of the color camera (DS-Fi3, Nikon Corp.) in TRUST are also provided as a reference.


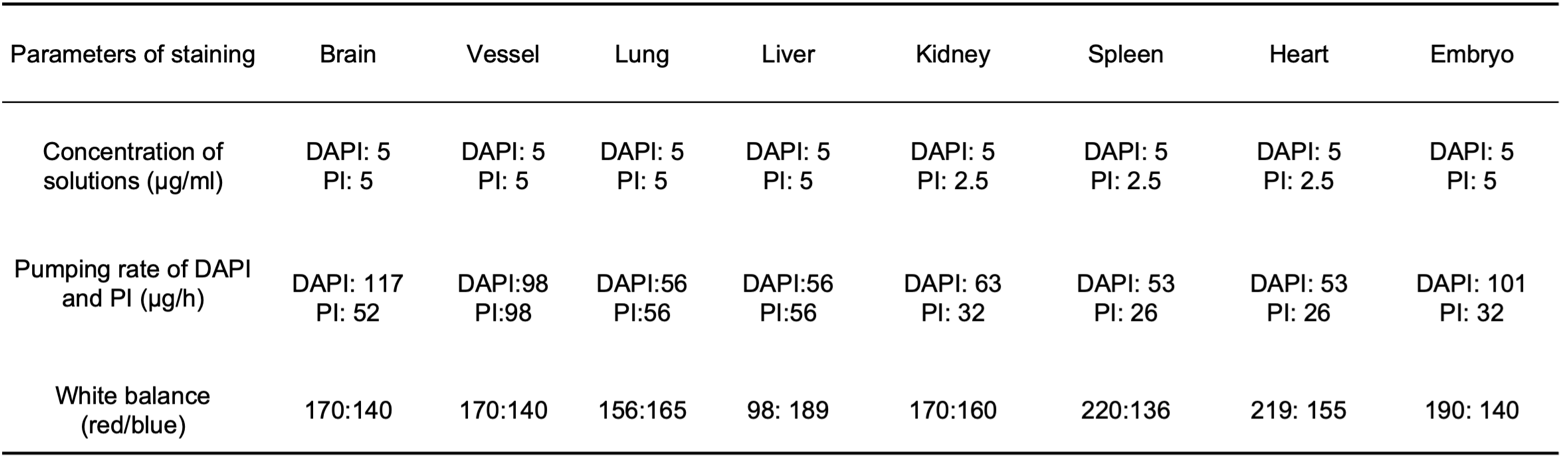


**Supplementary file 1b. Comparison of TRUST with other whole-organ imaging modalities.** The imaging speed of each modality is calculated based on the reported literature for whole mouse brain imaging, including serial two-photon tomography (STPT), microtomy-assisted photoacoustic microscopy (mPAM), micro-optical sectioning tomography (MOST), fluorescence micro-optical sectioning tomography (fMOST), light-sheet fluorescence microscopy (LSFM), and wide-field large-volume tomography (WVT).

**
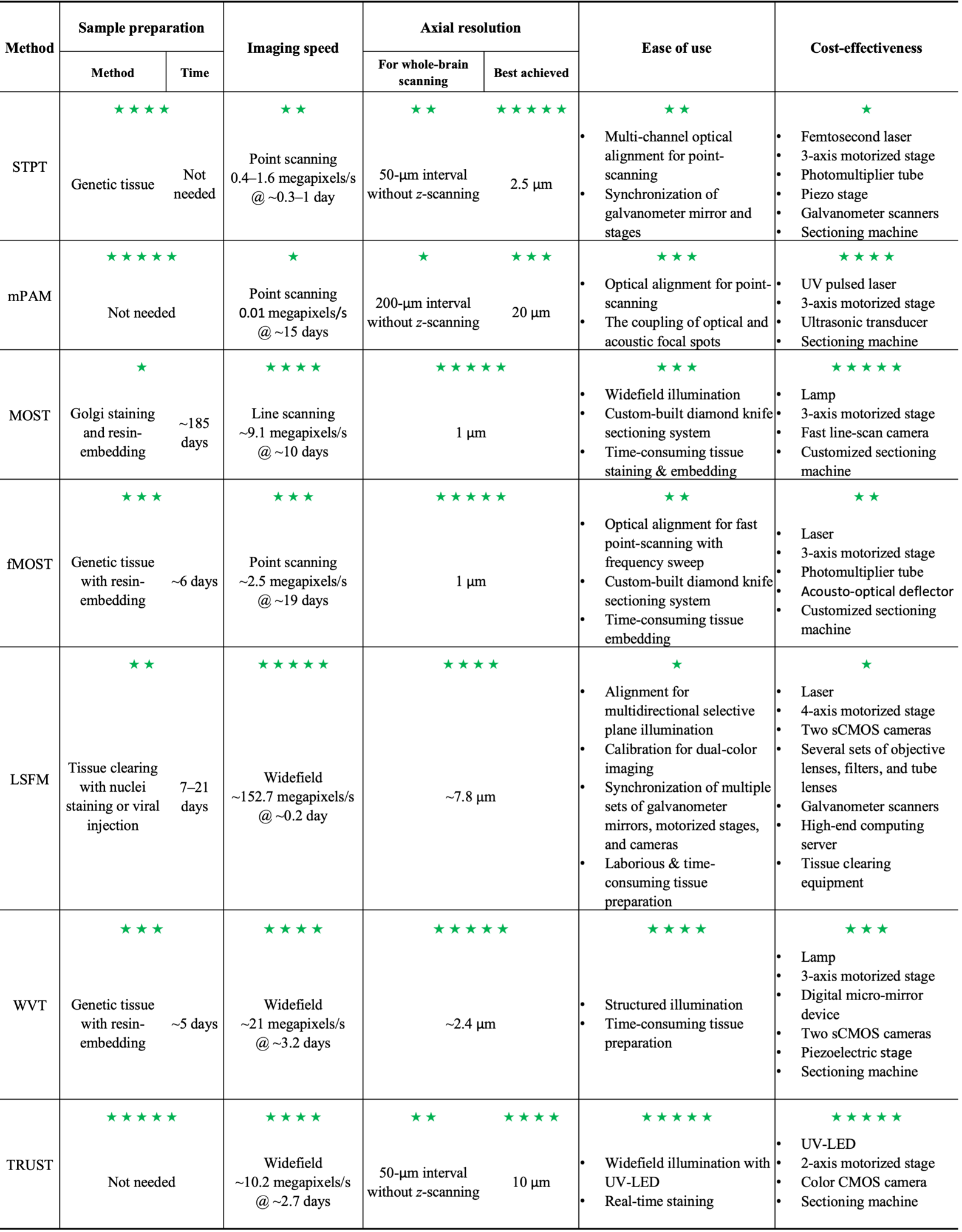
**

*Note: Imaging speed is defined by the ratio of the total number of pixels and the required time for whole-organ image acquisition. For multi-channel imaging systems, the number of pixels will be further multiplied by the number of channels. In STPT, depending on the XY sampling distance, the whole dataset (8.6×10^9^–1.3×10^11^ pixels) can be imaged within 6.5–24 hours. Then its imaging speed can be calculated as 0.4–1.6 megapixels/s. The imaging speed of mPAM has already been explicitly stated which is 0.01 megapixels/s. The imaging speed of MOST can be calculated based on the claim that the average acquisition rate was about 0.11 µs per voxel. The imaging speed of fMOST can be calculated based on the claim that the average acquisition rate was about 0.4 µs per voxel. In LSFM, the imaging speed was claimed as 0.5 terabytes (TB)/hour/color. Because the bit depth of images is 16, the imaging speed can then be converted as $\frac{0.5TB \times{1024}^{4}/(16bits/8 )}{1h*60*60}=76.35$ megapixels/s. The system supports dual-channel imaging, so the speed can be up to 152.7 megapixels/s. In WVT, it took ~77 hours to get the whole dataset (10.9 TB) which totally contains two channels. Because the bit depth of images in each channel is 16, the imaging speed of single channel can then be converted as $\frac{10.9TB/2 \times{1024}^{4}/(16bits/8 )}{77h*60*60}=$10.8 megapixels/s. Then, the speed of dual-channel imaging is 21.6 megapixels/s. TRUST took ~64 hours to get the whole dataset (~7.8 × 10^11^ voxels). Because there are three color channels (RGB), the imaging speed can then be calculated as $\frac{7.8 \times{10}^{11}}{64h*60*60}\times3=10.2$ megapixels/s. In STPT, mPAM, and TRUST, although a higher axial resolution has been demonstrated when imaging a relatively small region of the sample, a larger axial sectioning interval (50–200 µm) was applied when imaging the whole mouse brain.
